# Transcriptomically-Guided Pharmacological Experiments in Neocortical and Hippocampal NPY-Positive GABAergic Interneurons

**DOI:** 10.1523/ENEURO.0005-22.2022

**Published:** 2022-04-25

**Authors:** Sanne Beerens, Jochen Winterer, David Lukacsovich, Csaba Földy, Christian Wozny

**Affiliations:** 1 Strathclyde Institute for Pharmacy and Biomedical Sciences, University of Strathclyde, Glasgow, G4 0RE, United Kingdom; 2 Laboratory of Neural Connectivity, Brain Research Institute, University of Zürich, 8057 Zürich, Switzerland; 3 MSH Medical School Hamburg, Medical University, Institute for Molecular Medicine, 20457 Hamburg, Germany

**Keywords:** 5HT2a, GABAergic interneuron, neurogliaform cells, NPY, PatchSeq, Shisa9

## Abstract

Cortical GABAergic interneurons have been shown to fulfil important roles by inhibiting excitatory principal neurons. Recent transcriptomic studies have confirmed seminal discoveries that used anatomic and electrophysiological methods highlighting the existence of multiple different classes of GABAergic interneurons. Although some of these studies have emphasized that inter-regional differences may exist for a given class, the extent of such differences remains unknown. To address this problem, we used single-cell Patch-RNAseq to characterize neuropeptide Y (NPY)-positive GABAergic interneurons in superficial layers of the primary auditory cortex (AC) and in distal layers of area CA3 in mice. We found that more than 300 genes are differentially expressed in NPY-positive neurons between these two brain regions. For example, the AMPA receptor (AMPAR) auxiliary subunit Shisa9/CKAMP44 and the 5HT2a receptor (5HT2aR) are significantly higher expressed in auditory NPY-positive neurons. These findings guided us to perform pharmacological experiments that revealed a role for 5HT2aRs in auditory NPY-positive neurons. Specifically, although the application of 5HT led to a depolarization of both auditory and CA3 NPY-positive neurons, the 5HT2aR antagonist ketanserin only reversed membrane potential changes in auditory NPY-positive neurons. Our study demonstrates the potential of single-cell transcriptomic studies in guiding directed pharmacological experiments.

## Significance Statement

Using single-cell Patch-RNAseq, we characterized neuropeptide Y (NPY)-positive GABAergic interneurons in superficial layers of the primary auditory cortex (AC) and in dendritic layers of CA3. A few hundred genes were found to be differentially expressed in NPY-positive neurons between these two brain regions, including AMPA receptor (AMPAR) auxiliary subunit Shisa9/CKAMP44 and the 5HT2a receptor (5HT2aR). These findings guided us to perform pharmacological experiments that revealed a role for 5HT2aRs in superficial auditory NPY-positive neurons.

## Introduction

For decades, neurons were mainly characterized by their anatomic and electrophysiological properties, and marker expression (for review, see Klausberger and Somogyi, 2008; DeFelipe et al., 2013; Tremblay et al., 2016; Yuste et al., 2020). The identification of molecular markers in combination with sophisticated genetic tools such as the Cre-lox system has facilitated studies aimed at a detailed functional characterization of classes and subclasses of neurons (Tsien, 2016). More recently, single-cell sequencing techniques have enabled high-throughput studies that, for the first time, characterized the expression of thousands of genes in single cells (Zeisel et al., 2015; Tasic et al., 2016, 2018). Further, a combination of transcriptomic sequencing and brain slice single-cell electrophysiology known as Patch-RNAseq has been used to get a more refined picture of gene expression pattern and regional specificity (Cadwell et al., 2016; Földy et al., 2016; Fuzik et al., 2016).

One particular class of GABAergic interneurons has received our close attention: the neuropeptide Y (NPY)-expressing neurogliaform cells (Wozny and Williams, 2011; Webster et al., 2021). These cells have mainly been described in area CA1 (Price et al., 2005) and the somatosensory cortex (Tamás et al., 2003), but their existence in other brain regions remains unclear (Weiss and Veh, 2011; Webster et al., 2021). At least in the hippocampal area CA1, a closely related type of GABAergic neuron, called Ivy cells, has been identified (Fuentealba et al., 2008). Both neuroglia and Ivy cells express NPY but are considered two separate classes based on their anatomic position. Neurogliaform cells are mostly located in the vicinity of pyramidal dendrites in stratum lacunosum moleculare (SLM), whereas Ivy cells reside in the pyramidal cell layer of CA1 (Fuentealba et al., 2008; Capogna, 2011; Armstrong et al., 2012). Further, still in CA1, two different types of neurogliaform cells were identified based on whether or not they expressed neuronal nitric oxide synthase (nNOS; Tricoire et al., 2010). These two types are presumed to originate from different neurogenic origins: nNOS-expressing cells from the medial ganglionic eminence (MGE) and nNOS-negative cells from the caudal ganglionic eminence (CGE; Tricoire et al., 2010). In the neocortex, neurogliaform cells have been most thoroughly characterized in superficial layers of motor, visual and sensory areas (Kalinichenko et al., 2006; Chittajallu et al., 2013, 2020; Ibrahim et al., 2021).

Here, we aimed to use a comprehensive transcript-pharmacological approach to investigate NPY-expressing interneurons in the neocortical auditory and allocortical hippocampal CA3 region. These two regions are known to comprise of neurogliaform cells, but not much is known about their transcriptomic composition (e.g., whether or not they express neuroglia-associated markers, nNOS or neuron-derived neurotrophic factor (NDNF; Tasic et al., 2016), developmental origin (MGE or CGE), and pharmaco-physiological function. At least in the superficial layer of auditory cortex (AC), the importance of presumed neurogliaform cells in fear conditioning has been demonstrated (Letzkus et al., 2011), and also that these cells expressed NDNF (Abs et al., 2018). However, their transcriptomic composition has not been analyzed in detail (Kalish et al., 2020).

Using Patch-RNAseq on cells sampled from NPY-EGFP transgenic mice, we show differential expression of >300 genes between CA3 and auditory cortical NPY-positive cells. These included differential expression of neurodevelopmental markers, which suggested that auditory and CA3 NPY-expressing cells originated from CGE and MGE, respectively. Further, based on the transcriptomic insights, we investigated the role of a specific subtype of 5HT receptors (5HTRs) in pharmacological experiments, which revealed a role for 5HT2aRs in auditory cortical, but not in hippocampal NPY-positive interneurons.

In summary, our experiments highlight the benefit of performing targeted cell-specific and region-specific sequencing to guide pharmacological experiments.

## Materials and Methods

### Animals

The single-cell RNA sequencing experiments were conducted in Zurich, Switzerland. All animal protocols were approved by the Veterinary Office of Zürich Kanton. The University of Zurich animal facilities comply with all appropriate standards.

The pharmacological experiments were performed in Glasgow, United Kingdom. All procedures were conducted in accordance with the relevant United Kingdom legislation [the Animals (Scientific Procedures) Act, 1986]. Both in Zurich and in Glasgow, male and female mice from the NPY-hrGFP strain were used (van den Pol et al., 2009).

### Single-cell RNA sequencing

Samples were collected and processed as described previously (Földy et al., 2016; Lukacsovich et al., 2019; Winterer et al., 2019). In short, after sequencing, raw reads were de-multiplexed and preprocessed using Trimmomatic and Flexbar. Raw sequencing reads were aligned to the Ensembl GRCm38 reference transcriptome (version 2015-06-25), using STAR aligner with the following parameters: trimLeft = 10, minTailQuality = 15, minAverageQuality = 20, minReadLength = 30, “single-end/paired-end,” and “sense/antisense/both” options. Gene counts were calculated using HTSeq. For convenience, Ensembl gene IDs were converted to gene symbols using the mouse GRCm38 version 86 GTF file as a reference. In a few cases where different Ensembl gene IDs identified the same gene symbol, average gene counts were used.

For both quality control and normalization, we used scran (Lun et al., 2016). For each cell, we calculated the log of the number of unique genes that were detected, and removed cells with a value of at least 3.5 median absolute deviations (MAD) less than the median. For normalization, we used computeSumFactors with sizes of 10 and 20. Cells that had negative or zero size were removed. For further analysis and plotting, gene counts were converted into log2 space with a pseudo-count of 1.

### Electrophysiology and pharmacology

Brains were sliced in the horizontal plane for hippocampal recordings or the coronal plane for neocortical recordings as previously described (Winterer et al., 2019; Beerens et al., 2021). NPY cells were recorded from superficial layers [layer 1 (L1)-L2] of the AC and SLM of the CA3 in ACSF using standard potassium gluconate solutions [for the sequencing experiments: ACSF: 126 mm NaCl, 2.5 mm KCl, 10 mm glucose, 1.25 mm NaH_2_PO_4_, 2 mm MgCl_2_, 2 mm CaCl_2_, and 26 mm NaHCO_3_; intracellular solution: 95 mm potassium gluconate, 50 mm KCl, 10 mm HEPES, 4 mm Mg-ATP, 0.5 mm Na-GTP, and 10 mm phosphocreatine; pH 7.2, KOH-adjusted, 300 mOsm (Winterer et al., 2019), and for the pharmacological experiments: ACSF: 115 mm NaCl, 25 mm NaHCO_3_, 3 mm KCl, 1.25 mm NaH_2_PO_4_, 2 mm CaCl_2_, 1 mm MgCl_2_, 3 mm sodium pyruvate, and 10 mm glucose; intracellular solution 125 mm potassium gluconate, 10 mm HEPES, 6 mm KCl, 0.2 mm EGTA, 2 mm MgCl_2_, 2 mm Na-ATP, 0.5 mm Na-GTP, and 5 mm sodium phosphocreatine (Beerens et al., 2021)].

The following drugs were used: TCB-2, ketanserin tartrate, and serotonin hydrochloride (all Tocris).

To assess the responses to pharmacology a continuous current-voltage (IV) protocol was applied (range −200–350 pA; step size 50 pA). Current injections lasted 1 s with 9 s in between current injection steps. The total duration of recording was 30 min with 30 μm 5HT and 10 μm ketanserin being added to the perfusion system 2 and 12 min after start of the recording, respectively.

For TCB-2 recordings, baseline spontaneous activity was recorded for 2 min before 10 μm TCB-2 was added to the perfusion system and spontaneous activity was recorded for another 10 min. The IV protocol (range −100–300 pA; step size 25 pA) was recorded both before and 10 min after application of the drug to see its effect on resting membrane potential (RMP), input resistance (Rin), and spiking frequency. Spontaneous activity was always recorded at RMP with 0 pA injected and IV protocols were recorded with a current injection to reach a RMP of −70 mV to keep IV conditions the same throughout the recording.

L6 PFC pyramidal neurons were also recorded to test TCB-2 activity as previously shown (Tian et al., 2016). The same protocol was used as for AC NPY-positive neurons except for a TCB-2 concentration of 5 μm.

### Data analysis and statistics

The pharmacology experiments were analyzed using AxographX. RMP, Rin, and firing frequency were extracted from the recordings. The effect of TCB-2 on the RMP was determined by taking the average RMP of the IV recording at baseline or 10 min after drug application. The Rin was calculated by the reduction in membrane potential in response to a −100-pA injection. The number of spikes per current injection was counted to calculate the spiking frequency for every 25-pA step of positive current injection to create a frequency-current-curve (fI-curve).

Regarding the 5HT and ketanserin experiments, the RMP was determined for every trace within the 100 ms preceding the current injection, resulting in an RMP value for every 10 s of the recording. The first 4 min of the recording were used as a baseline. The effect of 5HT on the RMP was determined by averaging the RMP values of 1 min surrounding the peak of the 5HT effect. The peak was defined as where the RMP was the highest, which was typically around 14 min into the recording. The RMP after ketanserin application was determined by taking the average of the RMP values from the last minute of the recording. The relative effects of 5HT and ketanserin on RMP were calculated by taking the average RMP values after application and subtracting the average RMP baseline values for each cell.

Statistical analysis was performed and graphs were generated in GraphPad Prism. Statistical tests used were *t* test and two-way ANOVA followed by Bonferroni multiple comparison test. The threshold for statistical significance was set at *p* < 0.05. The following indications of statistical significance are used: *p < 0.05, **p < 0.01, ***p < 0.001.

### Data availability

Raw data are freely available under NCBI GEO #GSE193293.

## Results

### NPY-positive neurons in AC and CA3: electrophysiological properties

**Figure 1.**
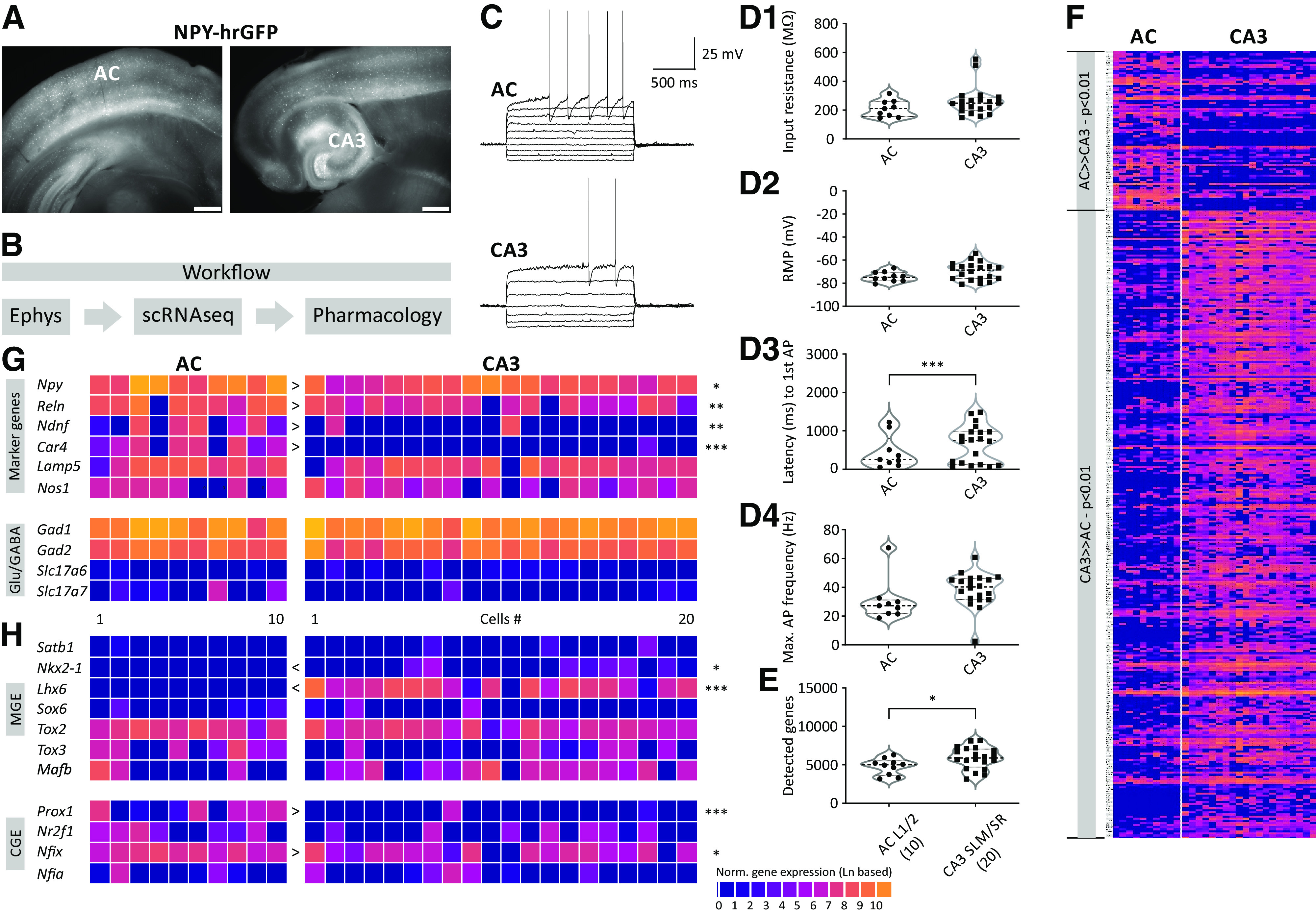
Single-cell transcriptomics of NPY-positive neurons in AC and CA3. ***A***, Overview image of a coronal neocortical (left) and a horizontal hippocampal (right) brain slice obtained from an NPY-hrGFP mouse. Scale bar: 1 mm. ***B***, Experimental flowchart starting with electrophysiology, followed by scRNAseq and pharmacological experiments. ***C***, Electrophysiological characterization of AC and CA3 NPY-positive neurons. Note the longer latency to action potential firing in CA3 neurons (see also ***D3***). ***D1–D4***, Active and passive electrophysiological properties of NPY-positive neurons. ***E***, Number of detected genes in AC and CA3 NPY-positive neurons. ***F*–*H***, Differentially expressed genes between AC and CA3 NPY-positive neurons.

We first recorded the electrical properties of NPY-positive neurons in L1 and L2 of the neocortical AC and in allocortical SLM at the boarder to stratum radiatum (SR) of area CA3 (Fig. 1*A*) in current-clamp using a 1.5-s current injection with increasing amplitude (range −200–350 pA; Fig. 1*C*) to confirm the typical late-spiking phenotype of neurogliaform cells (Overstreet-Wadiche and McBain, 2015). Then, the cytosol of NPY-positive neurons was aspirated via the glass pipette and a cDNA preparation was performed using established protocols (Földy et al., 2016; Lukacsovich et al., 2019; Winterer et al., 2019). In total 30 neurons, 10 from AC and 20 from CA3, passed our quality control criteria (see Materials and Methods).

With regard to electrophysiological properties, we analyzed passive (e.g., Rin and RMP; Fig. 1*D1*,*D2*, respectively) and active properties (e.g., action potential properties; Fig. 1*D3*,*D4*). None of these parameters were significantly different with the exception of the latency to first action potential (AP) (Fig. 1*D3*). CA3 NPY-positive neurons spiked significantly later than AC neurons using a near-rheobase current injection. In addition, we analyzed the sag potential in response to hyperpolarizing current injections, but this was not different between the two types (data not shown).

Together, our results show that the sampled neocortical and allocortical NPY-positive neurons were mostly similar with regard to their intrinsic electrical properties.

### Single-cell transcriptomics of NPY-positive neurons in AC and CA3

**Figure 2.**
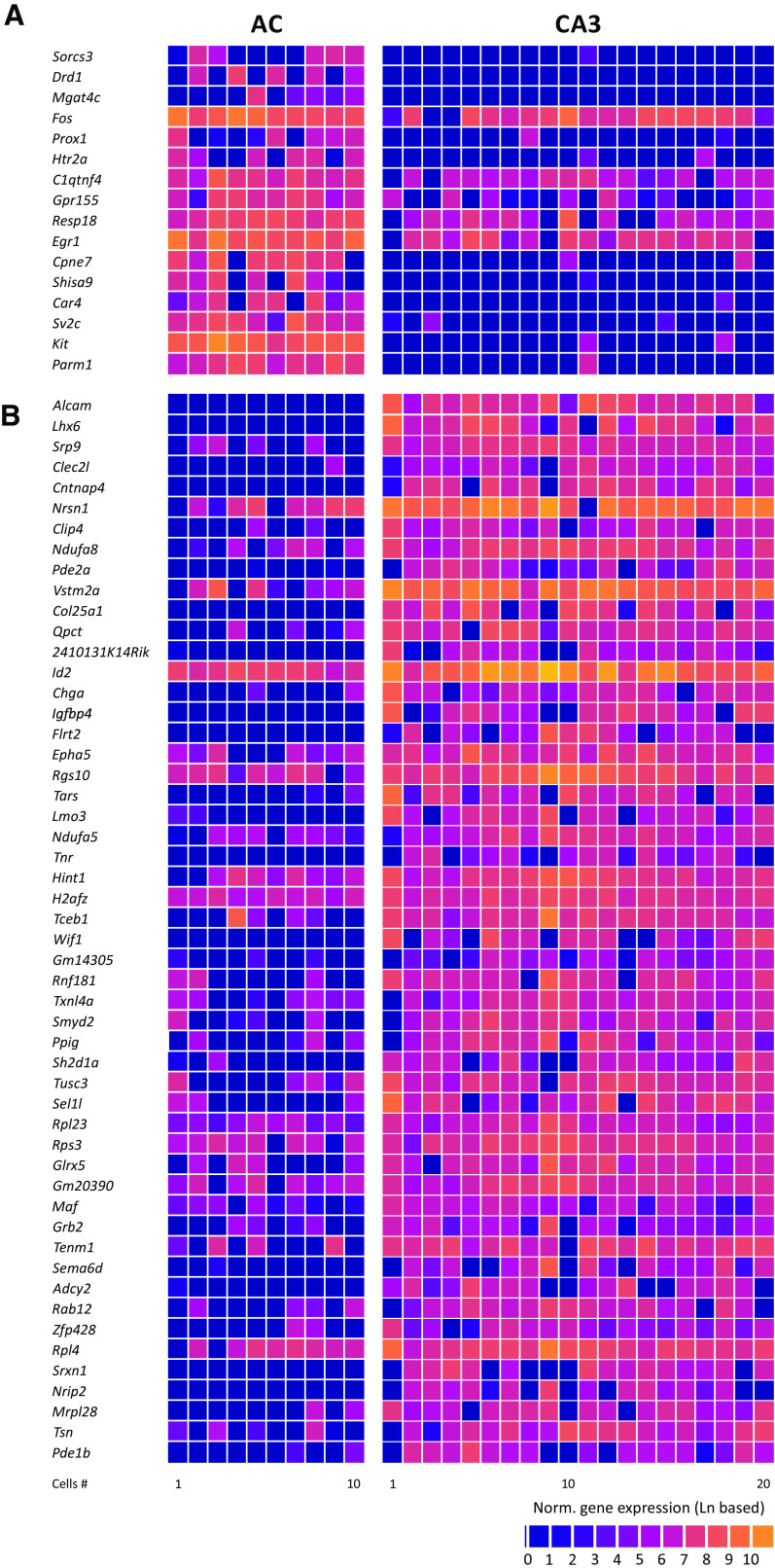
Differentially expressed genes with *p*-values < 0.001. ***A***, Genes that are significantly higher expressed in AC compared with CA3 neurons. Genes are displayed in the order of their statistical significance (from lower to higher; *Parm1*, *p* = 0.00000069). ***B***, Genes that are significantly higher expressed in CA3 compared with AC neurons. Genes are displayed in the order of their statistical significance (from higher to lower; *Alcam*, *p* = 0.0000082).

Molecularly, we found more pronounced differences between the two cell types. In AC and CA3 NPY-positive neurons, we detected 3163–6288 (4758 ± 334, mean ± SEM) and 3141–8149 (5877 ± 318, mean ± SEM; Fig. 1*E*, *p* = 0.0368, two-tailed *t* test) genes, respectively. While both types expressed the GABAergic markers *Gad1* and *Gad2* (Fig. 1*G*), over 300 genes are found to be differentially expressed in NPY-positive neurons between the two cell types (*p* < 0.01; Fig. 1*D*). Of these, >60 genes were differentially expressed with a *p*-value < 0.001 (Fig. 2).

As known markers, *Npy* and *Reln* were expressed in both types, but their expression was significantly higher in AC compared with the CA3 type. In line with previous studies, *Ndnf* and *Car4* were expressed in approximately half of the AC NPY-positive neurons (Tasic et al., 2016; Schuman et al., 2019), but only in two out of 20 CA3 NPY-positive neurons. Other markers, such as *Lamp5* and *Nos1* were not differently expressed in AC and CA3 NPY neurons (Fig. 1*G*).

Next, we examined the expression of transcription factors that are related to developmental origins (Lukacsovich et al., 2019; Winterer et al., 2019). MGE-derived interneurons are known to express the transcription factors *Lhx6, Nkx2-1*, *Satb1*, *Sox6*, *Tox2*, or *Tox3* (Batista-Brito et al., 2009; Paul et al., 2017; Lim et al., 2018). *Lhx6* and *Nkx2-1* were selectively expressed in CA3 NPY-positive neurons (Fig. 1*H*, *Lhx6*: *p* < 0.001 and *Nkx2-1*: *p* < 0.05). CGE-derived interneurons are known to express the transcription factors *Prox1*, *Nr2f1*, *Nfia*, or *Nfix* (Paul et al., 2017). Of these, *Prox1* and *Nfix* was selectively enriched in AC NPY interneurons (Fig. 1*G*, *Prox1*: *p* < 0.001; *Nfix*: *p* < 0.05), in which *Nr2f1* (also known as *Coup-TF1*) was also more frequently detected (Fig. 1*H*). By contrast, *Sox6* was rarely detected in either type (Fig. 1*H*).

### Differential expression of synaptic receptor coding genes

**Figure 3.**
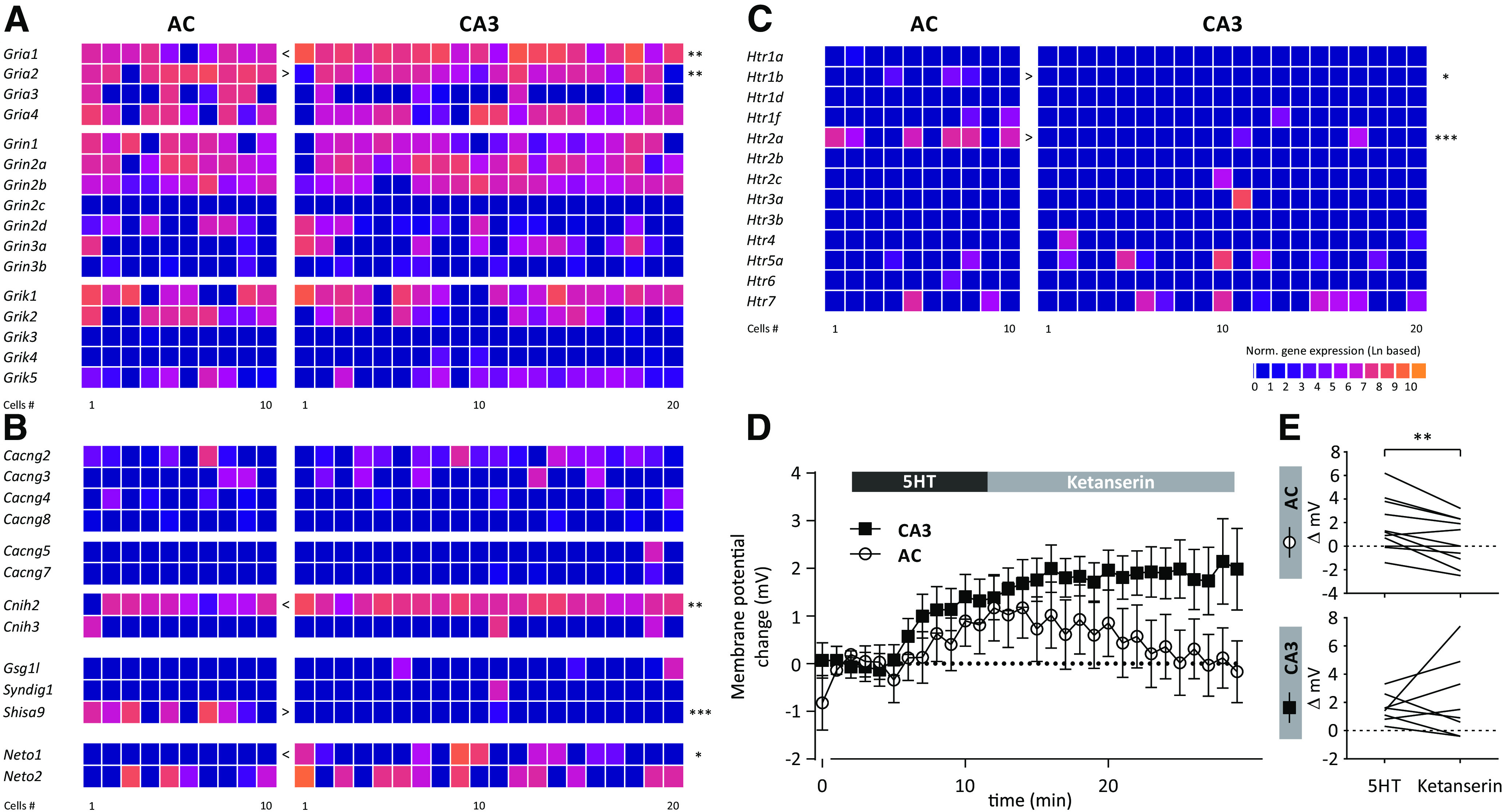
Transcriptomically-guided pharmacological experiments. Expression of iGluRs (***A***) and auxiliary subunits of iGluRs (***B***). ***C***, Expression of 5HTRs in auditory (AC) and CA3 NPY-positive neurons. ***D***, ***E***, Summary of changes in membrane potential following the application of 5HT and the 5HT2aR antagonist ketanserin.

We next explored the expression of ionotropic glutamate receptors (iGluRs) and their auxiliary subunits, because these play important roles in synaptic transmission and plasticity (Fig. 3*A*). L1 neocortical interneurons, for example, may use them to integrate information from other neocortical areas (D’Souza and Burkhalter, 2017). However, NMDA and kainate receptor were not differentially expressed (*Grin* and *Grik*, Fig. 3*A*).

By contrast, although AMPA receptor (AMPAR) subunits 1 and 2 (encoded by *Gria1* and *Gria2*) were expressed in both cell types, *Gria1* and *Gria2* were enriched in AC and CA3 NPY neurons, respectively (Fig. 3*A*).

Still regarding AMPARs, their auxiliary subunits have been shown to regulate the biophysical properties as well as the trafficking of AMPAR (Greger et al., 2017). Strikingly, the auxiliary subunit *Shisa9* (also known as CKAMP44) was predominantly expressed in AC, but not in CA3 NPY-positive neurons (*p* < 0.001; Fig. 3*B*). Given that this molecule has been shown to modulate short-term plasticity (von Engelhardt et al., 2010), it is plausible that AC NPY-positive neurons may display different responses following repetitive stimulation compared with CA3 NPY-positive neurons. In contrast to *Shisa9*, both *Cnih2* and *Neto1*, auxiliary subunits of AMPA and kainate receptors, respectively, were enriched in CA3 NPY-positive neurons (Fig. 3*B*). Taken together, NPY-positive neurons may assemble iGluRs and auxiliary subunits in a region-specific and/or developmental origin-specific fashion to achieve input-specific synaptic transmission.

### Transcriptomically-guided pharmacological experiments

It is well established that inhibitory GABAergic interneurons receive diverse neuromodulatory inputs (for review, see Pelkey et al., 2017), and are prominent targets of serotonin modulation (Athilingam et al., 2017; Winterer et al., 2019). In this domain, our transcriptomic analysis revealed surprisingly low overall expression of 5HTRs, with the exception of the *Htr2a* subunit, which was enriched in AC NPY-positive cells (*p* < 0.001; Fig. 3*C*).

5HT2a receptors (5HT2aRs) are G-protein-coupled receptors that have either been shown to form homomers (Brea et al., 2009) or heteromers (González-Maeso et al., 2008) mediating a wide range of physiological functions (Zhang and Stackman, 2015). To examine the functional consequences of *Htr2a* expression in AC NPY-positive neurons, we performed electrophysiological experiments testing pharmacological responses to the 5HT2aR antagonist ketanserin in both cell types. We applied 30 μm of 5HT (Fig. 3*D*) followed by the application of the 5-HT2aR antagonist ketanserin (10 μm). We found that 5HT depolarized both AC and CA3 NPY-positive interneurons, showing that functional HT receptors were present on both types (Fig. 3*D*,*E*). However, ketanserin only reversed these changes in AC, but not CA3, neurons. To further explore 5HT2a-mediated effects, we tested the 5HT2aR-specific agonist, TCB-2 (10 μm), on superficial AC NPY-positive neurons (*n* = 6 cells, *N* = 2 mice). However, TCB-2 alone did not alter the RMP, the Rin, and AP firing frequency measured as a frequency-current-curve (fI-curve range from −100 to 300 pA, step size 25 pA; data not shown). In order to test whether TCB-2 was effective, we applied it on prefrontal L6 pyramidal neurons, because these cells were recently shown to be responsive to 5 μm TCB-2 (Tian et al., 2016). However, in contrast to this previous report, we did not detect any response of TCB-2 (5 μm) on the above parameters during electrophysiological recordings from pyramidal cells (*n* = 7 cells, *N* = 3 mice; data not shown). As a consequence, we could not collect additional information on 5HT2aR function. Nonetheless, our pharmacological experiment with ketaserin (Fig. 3*D*,*E*) supported the functional expression of 5HT2aR in AC NPY-positive neurons.

## Discussion

Using single-cell transcriptomics we investigated mRNA expression of NPY-positive neurons in two different cortical regions, in the neocortical AC and allocortical area CA3 of the hippocampus (CA3). We found that gene expression pattern showed remarkable differences between these region, although NPY-positive neurons in both regions showed characteristic electrophysiological features of neurogliaform cells (Overstreet-Wadiche and McBain, 2015). Cells in both populations expressed well-known markers of neurogliaform cells such as *Reln* (reelin), *Lamp5*, and *Nos1*, but only superficial neocortical NPY-positive neurons expressed additional markers *Ndnf* and *Car4* (Tasic et al., 2018). However, depending on the statistical power applied, dozens to hundreds of genes were differentially expressed between AC and CA3 NPY-positive neurons.

### Developmental origin

Given that neurogliaform cells have been shown to originate from different neurogenic zones, MGE and CGE, it was first important to establish if, in addition to presumed regional differences, differences in the cells’ neurogenic origin could contribute to the observed discrepancies. In the cortex, it has been previously established that deep layer neurogliaform cells (L5 and L6) express the transcription factor *Lhx6*, and therefore these were considered to be derived from the MGE (*Lamp 5+ Lhx6+*), whereas the majority of superficial neurons were considered to be CGE-derived (Tasic et al., 2018; Gouwens et al., 2020). In the hippocampus, earlier single-cell PCR analyses already demonstrated a similar dichotomy in the origin of CA1 neurogliaform cells (Tricoire et al., 2010), which was significantly extended by a more recent large-scale transcriptomic study (Harris et al., 2018). This study analyzed the transcriptomic content of CA1 GABAergic interneurons and revealed the existence of 49 clusters that form 10 larger transcriptomic groups the authors called “continents.” Two of these were classified as neurogliaform cell containing continents. One consisted of presumed Ivy and MGE-derived neurogliaform cells, whereas the other consisted of CGE-derived neurogliaform cells (Harris et al., 2018). Cell numbers in these two larger groups were nearly evenly split. In our dataset from CA3, only two out of the 20 NPY-positive neurons did not express *Lhx6*, suggesting that CA3 NPY-positive cells are at least dominantly, and possibly entirely, derived from MGE.

### Auxiliary subunits of the AMPAR

Auxiliary subunits of the AMPAR have been described to be involved in trafficking of the AMPAR, but also in modulating the biophysical properties of the receptor complex (Greger et al., 2017). Among others, members of the Shisa family have been shown to bidirectionally modulate surface expression and AMPAR-mediated currents in a region-specific and cell type-specific manner (Abdollahi Nejat et al., 2021). Here, we provide further evidence for the latter notion by showing selective expression of Shisa9/CKAMP44 in AC NPY-positive neurons. With regard to potential consequences of Shisa9/CKAMP44 signaling, previous studies provided hints. Over-expression of Shisa9/CKAMP44 reduced short-term AMPA-receptor dependent plasticity in CA1 pyramidal neurons (von Engelhardt et al., 2010), whereas in dentate gyrus of Shisa9/CKAMP44 knock-out mice enhanced paired-pulse facilitation was observed (von Engelhardt et al., 2010). These would suggest that higher expression of Shisa9/CKAMP44 in AC NPY-positive neurons may weaken short-term plasticity of incoming synaptic inputs in these cells. Despite these insights, short-term plasticity properties of NPY-positive neurons remains mostly unknown. Future studies will have to address this issue and how this ties in with the neuronal firing properties of these neurons (Fuentealba et al., 2010; Li et al., 2014).

### Serotonin receptor signaling

Intriguingly, although 5HT3a receptor has been postulated as a specific marker of superficial neurons in the neocortex (Tremblay et al., 2016), we could not find evidence supporting this notion in cells collected from superficial AC. By contrast, we found that only AC, but not CA3, NPY-positive cells expressed 5HT2aR subunits. We followed up on this finding with transcriptomically-guided pharmacological experiments, which revealed functional modulation of 5HT responses by application of the 5HT2a antagonist ketaserin (Fig. 3), underscoring our transcriptomics-based finding. Previously, using transgenic mice, *Htr2a* expression was found in deep layer pyramidal neurons, but also in fast-spiking and delayed-spiking interneurons in all layers (Weber and Andrade, 2010). Fast-spiking cells likely represented parvalbumin (PV)-positive interneurons, whereas delay-spiking cells likely represented a diverse population of only partially neurogliaform cells. A different study has shown that 5HT2aR activation depolarizes PV-positive interneurons and increases their Rin (Athilingam et al., 2017). By contrast, only one third of all L5 pyramidal neurons in the prefrontal cortex responded, by increased AP firing, to the application of 5HT (Béïque et al., 2007). Similarly to 5HT, application of selective 5HT2 agonist αm-5-HT also induced AP firing in these cells (Béïque et al., 2007). While we tried application of another selective 5HT2 agonist, TCB-2, previously also shown to affect prefrontal L6 pyramidal neurons, we could not detect changes in AC NPY-expressing neurons by TCB-2 alone. Given that in our additional control experiments, TCB-2 did not induce AP firing in prefrontal cortical L6 pyramidal neurons either (not shown), these experiments remain inconclusive. However, our observation that the 5HT2a-specific antagonist ketanserin reversed the depolarization induced by 5HT in AC, but not in CA3, NPY-expressing cells, provided evidence for the functional expression of 5HT2a subunit-containing 5HTRs in AC NPY-expressing cells. This finding may have consequences to human clinical pharmacology. Antidepressant effects of psychedelics, such as psilocybin, are believed to manifest themselves through acting on 5HT2aRs (for review, see Nutt et al., 2020; Carhart-Harris et al., 2021). In this manner, our findings would implicate AC NPY-expressing cells as a cellular substrate of these antidepressant effects.

In summary, our study provides detailed insights into the transcriptomic composition of NPY-expressing neurons in the AC and hippocampal CA3 areas, and reveal modulatory effects by 5HT2a specifically on the AC population. Further, our study demonstrates how transcriptomically-guided pharmacological experiments can generate physiological knowledge. Expanding on this framework, future studies have the potential to facilitate the identification and understanding of functional, anatomic, molecular and pharmacological properties of neurons in a cell type-specific and region-specific manner, and augment transcriptomic brain cell atlases.

## Synthesis

Reviewing Editor: Arianna Maffei, Stony Brook University Decisions are customarily a result of the Reviewing Editor and the peer reviewers coming together and discussing their recommendations until a consensus is reached. When revisions are invited, a fact-based synthesis statement explaining their decision and outlining what is needed to prepare a revision will be listed below. The following reviewer(s) agreed to reveal their identity: NONE.

The reviewers agree that the manuscript provide an important contribution to the field and that the data are overall well presented and appropriately interpreted.

The reviewers asked for clarifications on several methodological and analytical approaches and raised a few concerns regarding variability that could potentially lead to overstating some conclusions.

The specific points raised by the reviewers follow below.

Reviewer #1

The authors present data from 30 Patch-Sequenced NPY neurons sampled from the hippocampus and auditory cortex. They follow up the gene expression data with e-Phys upon pharmacological administration of 5HT, 5HT2a-anatgonist and 5HT2a-agonist. This is an interesting study pairing Patch-Seq with pharmacological manipulations.

Major concern:

1> Line 38-39: The statement made in the abstract, “individual studies have rarely addressed regional specific differences in gene expression in a given subclass of neuron” is not true. In fact, Tasic et. al. 2018 paper titled “Shared and distinct transcriptomic cell types across neocortical areas” explores this very issue in Fig 2b (left panel) showing there are more genes differentially expressed between equivalent glutamatergic neuron clusters than equivalent GABAergic neuron clusters between brain areas ALM and VISp. Hence this statement should be removed or at the least rectified to acknowledge existing work.

2> Sample size is low but given Patch-seq is more labor-intensive this is understandable. However, authors should show the cell-cell variability of expression for given transcripts, as aspirated cytoplasmic content is highly-variable and is a known concern for this method.

3A> Fig 1 H, while 3 transcription factors were shown for MGE lineage only Lhx6 is consistently expressed, authors should show expression of Sox6, Mafb, Tox3, and Tox2 as those are also MGE restrictied.

3B> Fig1H, Sox6 is listed as a CGE marker, in fact Batista-Brito 2009, Neuron showed it is expressed downstream of Lhx6 in post-mitotic interneurons from MGE. This should be rectified and Sox6 should be grouped with MGE. For CGE lineage authors can also show expression of transcription factors Sp8, Sall1, Nfix and Nfia.

4> Sst;Nos1 cells from the frontal cortex have been extensively sequenced by Paul et. al. 2017 titled “Transcriptional Architecture of Synaptic Communication Delineates GABAergic Neuron Identity”. These cells were exclusively Npy+, this manuscript would benefit from contrasting the AC and Hippocampal Nos1 gene signatures with frontal cortex and providing the reader with additional comparison.

Minor concerns

1> Chodl was shown to be restricted to Nos1 neurons by Tasic 2018 and Paul 2017 which are Npy+, it would be interesting to see if there is concordance in Chodl expression in AC and Hipp regions.

2> Most of the expression data is presented as heatmaps. Heatmaps are better suited for large numbers of samples or the grouping of many samples. Here sampling size being much lower and genes discussed being smaller showing box plots/violin plots would be a better and more intuitive visualization for the reader.

Reviewer #2

The authors describe a Patch-seq analysis of NPY interneurons of the auditory cortex and the CA3 to characterize regional specific differences in gene expression of this interneuron subpopulation. The manuscript highlights the difference at the level of single genes between NPY neurons of the two areas. Based on the differential expression of the 5HT2a gene, the study explores the role of this receptor in NPY neurons of the auditory cortex indicating functional modulation of this receptor by the receptor antagonist ketaserin.

The data presented in the manuscript is interesting and provide new insights into the transcriptome of interneurons subpopulation, defining their regional specific profile. However, the single gene analysis proposed in the manuscript is limited and does not make use of the full potential of single cells transcriptomics data. Below a few comments that I would like to see addressed by the authors:

1. Please provide a detailed account of the scRNA sequencing data analysis, including controls and normalization method.

2. The study would benefit from a more sophisticated analysis of the sequencing data. It is not clear why the authors did not exploit the opportunity to map the Patch-seq profile to an existing transcriptome reference atlas. This will allow to classify the Patch-seq samples and characterize more accurately the transcriptomic identification of the cell type, strengthening the idea of a regional diversification. Furthermore, the study will benefit from multimodal data exploration and visualization. Several methods have been recently developed that allow for visualization of gene expression and electrophysiological properties.

3. It would be interesting to see morphological data on the samples used for Patch-seq, to relate the differential expression of the two populations with potential morphological difference. If not available, the authors should at least discuss this issue in the Discussion.

4. Please provide the original transcriptome data, either directly or through a dedicated database.
